# Strain‐Specific Yeast Polysaccharides Enhance Growth, Antioxidant Capacity, Immune Response and Gut Microbiota Homeostasis in Red Tilapia Cultured Under Seawater Conditions

**DOI:** 10.1155/anu/3589937

**Published:** 2026-05-12

**Authors:** Ruimin Yuan, Xiaoyi Wu, Juyun He, Zhiyu Zhou

**Affiliations:** ^1^ School of Life and Health Sciences, Hainan University, Haikou, 570100, Hainan, China, hainu.edu.cn; ^2^ Hainan Provincial Key Laboratory for Tropical Hydrobiology and Biotechnology, Hainan University, Haikou, 570228, Hainan, China, hainu.edu.cn; ^3^ School of Marine Biology and Fisheries, Hainan University, Haikou, 570100, Hainan, China, hainu.edu.cn; ^4^ School of Pharmaceutical Sciences, Guangzhou Medical University, Guangzhou, 511436, China, gzhmc.edu.cn

**Keywords:** gut microbiota homeostasis, marine aquaculture, red tilapia, Saccharomyces cerevisiae, yeast polysaccharides

## Abstract

This study evaluated the antibacterial activity and functional effects of yeast polysaccharides derived from three *Saccharomyces cerevisiae* strains (L6, L9, and L10) on growth, immunity, and gut health of red tilapia cultured under seawater conditions. An in vitro antibacterial assay against *V. parahaemolyticus* and *V. vulnificus* was conducted, followed by a 16‐week feeding trial using six isonitrogen (crude protein: 370 ± 8 g kg^−1^) and iso‐energy (gross energy: 11.4 ± 0.2 MJ kg^−1^) diets: control, commercial β‐glucan, MOS, L6, L9, and L10, each at 1 g/kg. A total of 720 healthy fish (20.06 ± 0.06 g) were randomly assigned to 24 floating cages (1 × 1 × 1 m; four replicates per treatment). Results showed that all YPS exhibited antibacterial activity against *Vibrio spp*., with L10 showing the strongest inhibition in vitro. Fish fed L10 also exhibited the highest weight gain rate (WG) and lowest feed conversion ratio (FCR) compared with the control group (*p*  < 0.05). Plasma biochemical indices indicated that the L10 group significantly increased total antioxidant capacity, and lysozyme while decreasing malondialdehyde (*p*  < 0.05). Meanwhile, the intestinal anti‐inflammatory cytokines were significantly upregulated, and the proinflammatory cytokines were significantly downregulated with the inclusion of yeast polysaccharide derived from the L10 strain (*p*  < 0.05). Furthermore, L10 also improved intestinal morphology and barrier integrity while modulating the microbiota toward beneficial taxa, characterized by increased *Cetobacterium* and reduced *Photobacterium*. Integrated transcriptomic and metabolomic analyses further revealed activation of immune‐, metabolism‐, and homeostasis‐related genes (e.g., *PAPSS2*, *rhbg*, and *CBS*), alongside the enrichment of amino acids and microbial metabolites (e.g., indole derivatives) associated with mucosal immune regulation. Overall, this study revealed that supplementation of 1 g/kg L10 yeast polysaccharide can promote growth, antioxidant capacity, immune homeostasis, and gut microbiota balance in red tilapia, highlighting the potential as next‐generation functional feed additives for sustainable marine aquaculture.

## 1. Introduction

Aquaculture serves as a critical approach to sustainably meeting the global protein demand [1]. Due to the environmental pressures and limitations faced by traditional freshwater aquaculture, efficient and environmental‐friendly mariculture has become increasingly important [2]. However, to date, the diversity of species suitable for marine ranching remains limited, which seriously restricts the sustainable development of the marine ranching system. Red tilapia (*O. niloticus* × *O. mossambicus*) has attracted wide attention because of its rapid growth, strong adaptability, tolerance to environmental stress, and broad consumer acceptance [3, 4]. Consequently, Ocean Ranch Red Bream (HD Bream)—a selectively bred red tilapia strain characterized by high seawater adaptability, rapid growth, and production efficiency—has been successfully developed by the Haid Group. Despite its potential for marine ranching, limited research has investigated the optimal physiological and nutritional responses of HD bream under full‐seawater conditions.

Meanwhile, the long‐term intensive farming model of red tilapia exacerbates challenges such as oxidative stress, inflammation, and intestinal barrier dysfunction, which collectively increase disease susceptibility and reduce feed efficiency, growth performance, and profitability [5]. Vibriosis, particularly infection caused by *Vibrio parahaemolyticus*, represents one of the most severe bacterial diseases threatening fish health and aquaculture productivity [6]. This highly virulent pathogen produces toxins that cause intestinal tissue damage, systemic infection, and acute mortality [7]. Frequent outbreaks of vibriosis in intensive tilapia farming have led to substantial economic losses in China, highlighting the urgent need for effective and environmentally friendly alternatives to antibiotics.

Probiotics, prebiotics, postbiotics, and synbiotics are recommended as promising alternatives. Among these strategies, yeast‐based products—particularly those derived from *Saccharomyces cerevisiae*—have been extensively investigated and applied in aquaculture due to their safety, stability, and multifunctional bioactivities [8]. Increasing evidence suggests that polysaccharides—mainly derived from plant cell walls, agricultural waste, seaweeds, and microorganisms—possess considerable potency in the prevention and control of bacterial diseases such as vibriosis [9–11]. Among them, yeast‐derived polysaccharides (YPS) have been identified as promising bioactive compounds with diverse functional properties [12]. Yeast cell wall polysaccharides, mainly containing β‐glucans and mannoproteins, are increasingly recognized as functional feed additives in aquaculture owing to their versatile bioactivities [13]. In tilapia, dietary supplementation with β‐glucans and mannan‐oligosaccharides (MOS) has been widely reported to effectively promote growth performance [14–18]. Beyond growth promotion, YPS—either alone or in combination—has demonstrated protective effects against chemical stressors such as fipronil, trichlorfon, and copper [19–21] and have been shown to enhance resistance to bacterial pathogens including *Streptococcus agalactiae*, *Streptococcus iniae*, *Aeromonas hydrophila*, and *Flavobacterium columnare* [14, 22–24]. Additionally, dietary MOS supplementation has been reported to improve flesh quality and alleviate the adverse impacts of high‐carbohydrate diets on growth [25, 26], while moderate inclusion levels (1.5 g kg^−1^ feed) has been found to promote gonadal development and enhance reproductive performance in red tilapia broodstock [27]. Despite these promising outcomes, most previous investigations have focused on short‐term effects of YPS supplementation on juvenile fish, leaving significant knowledge gaps regarding their long‐term effects on physiological homeostasis and microbial community dynamics.

To address these knowledge gaps, the present study evaluated the long‐term effects of YPS obtained from different *Saccharomyces cerevisiae* strains as functional feed additives. Growth performance, antioxidant capacity, immune and inflammatory responses, intestinal morphology, and gut microbiota composition were systematically assessed in HD Bream reared under seawater conditions. Overall, the findings are expected to provide new insights into the strain‐specific functional properties and potential mechanisms of YPS. This study also offers a scientific basis for the rational selection of effective and sustainable YPS sources for application in red tilapia aquaculture.

## 2. Materials and Methods

### 2.1. Strain Selection and Yeast Polysaccharides Preparation

#### 2.1.1. Strain Isolation and Maintenance

Candidate yeast strains were isolated from marine sediments collected in Yangjiang, Guangdong, China. The isolates were cultured in yeast peptone dextrose (YPD) broth containing 1% yeast extract, 2% peptone, and 2% dextrose at 28°C with shaking at 180 rpm for 24 h. Strains were maintained on YPD agar (YPD supplemented with 2% agar) at 4°C and subcultured biweekly. For long‐term preservation, cultures were stored in 20% (v/v) glycerol at −80°C.

#### 2.1.2. Vibrio‐Binding Assay

The binding capacity of yeast strains was assessed against *Vibrio parahaemolyticus* (VP32) and *V. vulnificus* (VP44) using a live‐cell adsorption assay. VP32/VP44 cultures were adjusted to ~1 × 10^8^ CFU/mL (OD600 = 0.8 ± 0.05). Yeast suspensions (OD600 = 1.0 ± 0.05, fivefold dilution) were mixed with the bacterial suspension and incubated at 28°C with gentle shaking (100 rpm). OD600 was recorded at 0 and 1.5 h, and the binding rate was calculated as the percentage decrease in OD600. Strains L6, L9, and L10 exhibited significantly higher binding rates (*p* < 0.05) than the others and were selected for subsequent polysaccharide preparation.

#### 2.1.3. Yeast Polysaccharides Extraction

Yeast cells were harvested, washed, and disrupted on ice by bead‐milling using 0.5 mm glass beads. The disruption protocol consisted of six cycles with strain‐specific parameters: for L6 and L9, each cycle comprised 90s of disruption followed by 20 s of rest; for L10, each cycle comprised 40 s of disruption followed by 20 s of rest. The resulting suspensions were then hydrolyzed with cellulase (from *Trichoderma reesei*, ≥1000 U/g) and β‐glucanase (from *Aspergillus niger*, ≥500 U/g) at 50 U/mL each and incubated at 50°C for 4 h. The hydrolysates were freeze‐dried for 24 h, producing polysaccharide preparations with solubility exceeding 27.6%.

#### 2.1.4. Antibacterial Activity Assay

The antibacterial activity of the polysaccharide (purity ≥ 75%) was evaluated against VP32 and VP44. Bacterial suspensions (1 × 10^8^ CFU/mL) were incubated with polysaccharides (0.1%, w/v) at 30°C for 60 min. Controls included bacterial suspensions without polysaccharides and PBS alone. Following incubation, samples were serially diluted, plated on LB agar, and incubated at 37°C for 24 h. Antibacterial activity was expressed as the percentage reduction in CFU relative to the bacterial control (BC).

### 2.2. Experimental diets

Six isonitrogen (crude protein: 370 ± 8 g kg^−1^) and iso‐energy (gross energy: 11.4 ± 0.2 MJ kg^−1^) diets were formulated (Table 1), consisting of a control diet and five experimental diets (Y, S, L6, L9, and L10). The control diet contained no additive, while the experimental diets included two commercially available functional polysaccharides, S (a commercial mannan oligosaccharide product, ≥70% MOS) and Y (a commercial yeast β‐glucan product, ≥80% β‐glucan), which were purchased and included at the dose of 1 g/kg. The remaining three experimental diets were supplemented with yeast polysaccharides (1 g/kg) from strain L6, L9, and L10, respectively.

**Table 1:** Ingredients and proximate analysis of the experimental diets.

Ingredients (g/kg)	C	Y	S	L6	L9	L10
Corn protein meal^1^	90	90	90	90	90	90
Soybean meal (43%)^1^	420	420	420	420	420	420
Rapeseed meal^1^	100	100	100	100	100	100
Corn germ meal^1^	50	50	50	50	50	50
Rice bran	44.8	43.8	43.8	43.8	43.8	43.8
Wheat	190	190	190	190	190	190
Soybean oil	34	34	34	34	34	34
Soy lecithin	30	30	30	30	30	30
Calcium monobasic phosphate	20	20	20	20	20	20
Premix^2^	15	15	15	15	15	15
L‐Lys HCl (98.5%)	2	2	2	2	2	2
Choline chloride (50%)	1.5	1.5	1.5	1.5	1.5	1.5
Salt	2	2	2	2	2	2
Ethoxyqueline	0.2	0.2	0.2	0.2	0.2	0.2
Calcium propionate	0.5	0.5	0.5	0.5	0.5	0.5
Polysaccharides – Y	/	1	/	/	/	/
Polysaccharides – S	/	/	1	/	/	/
Polysaccharides – L6	/	/	/	1	/	/
Polysaccharides – L9	/	/	/	/	1	/
Polysaccharides – L10	/	/	/	/	/	1
Proximate analysis (dry basis, analyzed level)						
Crude protein (%)	37.54	36.94	36.13	36.76	36.20	36.28
Crude lipid (%)	9.91	9.80	10.56	10.32	10.11	9.81
Crude ash (%)	7.76	7.64	7.65	7.53	7.53	7.54
Energy (MJ kg)	11.4	11.2	11.3	11.6	11.4	11.3

*Note*: The compositions of premix include: Vit A, 3,800,000 IU/kg; Vit B_1_, 4200 mg/kg; Vit B_2_, 15,800 mg/kg; Vit B_6_, 6200 mg/kg; Vit B_12_, 36 mg/kg; Vit C, 87,500 mg/kg; Vit D_3_, 3,000,000 IU/kg; Vit E, 40,400 mg/kg; Vit K_3_, 9900 mg.kg; D‐calcium pantothenate, 20,200 mg/kg; nicotinamide, 25,500 mg/kg; folic acid, 2600 mg/kg; D‐biotin, 52 mg/kg; inositol, 36,000 mg/kg; K, 25,200 mg/kg; Na, 65,000 mg/kg; Mg, 26,300 g/kg; Zn, 30,000 mg/kg; Mn, 20,000 mg/kg; Cu, 5000 mg/kg; Fe, 150,000 mg/kg; Co, 400 mg/kg; and I, 4000 mg/kg; Se, 100 mg/kg.

^1^Corn protein meal and other major protein sources were purchased from Haid feedstuffs Co., Ltd. (Guangzhou, China).

^2^Premix was obtained from Hinter Environmental Technology Co., Ltd. (Guangzhou, China).

All raw ingredients were ground, sieved, and mixed according to the formulation. The mixtures were processed using a twin‐screw extruder (62‐type, Beijing Yanggong Co., Ltd.) with a pellet size of 1.5 mm, dried in a ventilated oven, coated with oil, and stored in sealed bags at 4°C until use. Proximate composition was analyzed following AOAC procedures [28], and gross energy was determined using an oxygen bomb calorimeter (Staufen, Germany).

### 2.3. Feeding Trial

Red tilapia were reared in outdoor ponds at the Yangjiang base of the Haid group (Guangdong, China). Before the feeding trial, fish were acclimated on a commercial diet (crude protein, 34%; crude lipid, 9.2%) for 2 weeks. After 24 h of fasting, 720 healthy fish (20.06 ± 0.06 g) were randomly allocated into 24 floating cages (1 × 1 × 1 m). Each cage was stocked with 30 fish, with 4 cages assigned to each treatment. To ensure uniformity, 30 fish of similar size were batch‐weighted at the start of the experiment. Both cage distribution and fish selection were randomized.

Fish were hand‐fed to apparent satiation twice daily (7:30 and 16:00) for 16 weeks. Water quality was monitored around the cages using a portable water quality meter (Sunpu Test, Beijing Sunpu Biochem. Tech. Co., Ltd., Beijing, China). During the trial, water temperature ranged from 23.2°C to 28.5°C, dissolved oxygen remained above 6.5 mg/L, pH was maintained at 7.5, nitrite and ammonia nitrogen concentrations were consistently below 0.01 mg/L and 0.1 mg/L, respectively; and salinity ranged between 20 and 25 ppm.

All animal experiments were approved by the Experimental Institution Animal Care and Use Committee at Hainan University (Approval Code: HNUAUCC‐2023‐00048).

### 2.4. Sample Collection and Laboratory Analyses

#### 2.4.1. Sample Collection

At the end of the feeding trial, fish were fasted for 24 h before sampling. The total biomass and number of fish in each cage were recorded. Seven fish per cage were randomly sampled: two were allocated for whole‐body composition analysis and the remaining five were designated for biochemical and molecular analyses. Fish were anesthetized using MS‐222 (100 mg/L). Blood was collected and centrifuged (3000 rpm, 10 min, 4°C) to obtain plasma. The intestinal tissues of the same five fish were dissected and partitioned for subsequent analyses, including gene expression profiling, histological examination, and intestinal microbiota characterization, and transcriptomic and metabolomic sequencing. All samples were immediately snap‐frozen in liquid nitrogen and stored at −80°C until further processing.

#### 2.4.2. Plasma Biochemistry

After being removed from the −80°C freezer and thawed at room temperature, serum samples were used to measure plasma biochemical parameters, including total cholesterol (TC), triglycerides (TGs), glucose (Glu), and alkaline phosphatase (AKP), using a BS‐400 automatic biochemical analyzer (Mindray, Shenzhen, China) with manufacturer‐supplied commercial kits. Antioxidant and immune indices, including malondialdehyde (MDA), total antioxidant capacity (T‐AOC), lysozyme (LYZ), and complement components C3 and C4, were assessed using assay kits from Jiancheng Bioengineering Institute (Nanjing, China). Additionally, the activities of D‐lactate (D‐lac) and diamineoxidase (DAO) in serum were qualified using commercial ELISA kits (Shanghai Bio‐Technology Co., Ltd.) following the manufacturer’s protocols.

#### 2.4.3. Gene Expression

Total RNA was isolated from the hindgut using E.Z.N.A. Total RNA Kit I (Omega Bio‐Tek, Norcross, Georgia) and was followed by purification with DNase I to remove genomic DNA contamination. RNA quality was assessed with a NanoDrop One spectrophotometer (Thermo Fisher Scientific): only RNA with an A260/A280 ratio of 1.8–2.0 and A260/A230 ratio ≥2.0 was used for subsequent experiments. RNA integrity was further verified by 1% agarose gel electrophoresis (clear 28S and 18S rRNA bands, no smearing). Subsequently, 1 µg of qualified total RNA was reverse‐transcribed to cDNA using the RR047A‐PrimeScript RT Reagent Kit (TaKaRa Bio Inc., Japan) following the manufacturer’s protocol (37°C for 15 min and 85°C for 5 s). qPCR was carried out on a CFX96 Real‐Time PCR System (BIO‐RAD) under the following thermal cycling conditions: initial denaturation at 95°C for 30 s, followed by 40 cycles of denaturation at 95°C for 5 s, annealing at 58 ± 2°C (optimized for each primer pair) for 30 s, and extension at 72°C for 30 s. A melting curve analysis (65–95°C, 0.5°C increment per s) was performed after qPCR to confirm the specificity of the amplification products (single peak). Relative gene expression was quantified by the 2−ΔΔCt method, with beta‐actin (*β-actin*) and elongation factor 1 α (*ef1α*) as the reference genes. Primer sequences of interleukin 1 beta (*il-1β*), interleukin 6 (*il-6*), interleukin 8 (*il-8*), interleukin 12 (*il-12*), tumor necrosis factor‐alpha (*tnf-α*), transforming growth factor‐beta 1 (*tgf-β1*), and interleukin 10 (*il-10*) are listed in Table 2. The standard curve was used for validation.

**Table 2:** Primer sequences for qPCR.

Gene name	Sequence (5′–3′)	Accession number/reference
*il-1β*	F‐GAGCACAGAATTCCAGGATGAAAG	XM_019365842.2
	R‐TGAACTGAGGTGGTTCCAGCTGT	
*il-6*	F‐ACAGAGGAGGCGGAGATG	XM_019350387
	R‐GCAGTGCTTCGGGATAGAG	
*il-8*	F‐ACAGTTACAACAGCTGGAATACAT	XM_019353306.2
	R‐CAGGGTCCCAAACACAATCAG	
*il-10*	F‐CAGCAGCAGGAGCATCAGCATT	KP645180.1
	R‐CACAGGAGGACGGTCTGAGAAGT	
*il-12*	F‐AGGTCAGCCAACTCGTGCCACT	XM_003437924.4
	R‐CCGTGATGTTCTGGAGCAGTGTTC	
*tnf-α*	F‐CTGAAGCACTAAAGGCGAAGAAACA	NM_001279533.1
	R‐TTTCTAGATGGATGGCTGCCTTG	
*tgf-β1*	F‐AAGAGGAGGAGGGAATACTTTGCCA	XM_025897821.1
	R‐GAAGCTCATTGAGATGACTTTGGG	
*β-actin*	F‐AGCCTTCCTTCCTTGGTATGGAAT	XM_003443127.5
	R‐TGTTGGCGTACAGGTCCTTACG	
*ef1*α	F‐ACATCGCTTGCAAGTTCAGC	MM_001279647.1
	R‐ACTTGATGACACCGACAGCC	

*Note*: *il-1β*, interleukin 1β; *il-6*, interleukin 6; *il-8*, interleukin 8; *il-10*, interleukin 10; *il-12*, interleukin 12; *tnf-α*, tumor necrosis factor‐alpha; *tgf-β1*, transforming growth factor‐beta; *β-actin*, beta‐actin; and *ef1α*, elongation factor 1 α.

#### 2.4.4. Histology

Intestinal samples (8 fish per treatment) were fixed in 10% neutral buffered formalin, dehydration through graded ethanol, cleared in xylene, and embedded in paraffin. Sections (5 μm) were cut with a microtome (Leica Biosystems, Germany) and stained with hematoxylin and eosin (H&E) following Bancroft and Gamble. Slides were examined under a light microscope (CX21, Olympus, Japan) equipped with a digital camera (Motic MC 2000) at 20x magnification. Villus height, villus width, crypt depth, and muscularis thickness were measured in five randomly selected fields per fish using ImageJ software v.154d (NIH).

#### 2.4.5. Intestinal Microbiota Analysis

Microbial analysis was conducted by Guangzhou Gene Denovo Biotechnology Co., Ltd. Total bacterial DNA was extracted with the Power Soil DNA Isolation Kit (MO BIO Laboratories, USA). The V3–V4 region of the 16S rRNA was amplified (forward primer, 5’‐ACTCCTACGGGAGGCAGCA‐3’; reverse primer, 5’‐GGACTACHVGGGTWTCTAAT‐3’) and sequenced on an Illumina Hiseq platform. Sequences with ≥97% similarity were clustered into OTUs. Representative sequences were aligned against the reference database to assign taxonomy at different levels. QIIME was used to generate abundance tables, and the Venn diagram was produced with the R package *VennDiagram*. Group differences were assessed with Metastats. Alpha diversity indices (Chao1, ACE, Shannon, and Simpson) and beta diversity were calculated, as well as sequencing coverage.

#### 2.4.6. RNA‐Seq Analysis

RNA‐seq analysis of intestine samples was performed using the Illumina Novaseq 6000 platform (Majorbio, China). Briefly, total RNA was extracted with TRIzol (Invitrogen, Carlsbad, CA, USA), quantified, and assessed for purity using a Nanodrop2000 spectrophotometer (Thermo Fisher, USA), and libraries were prepared using the Illumina TruSeq RNA Sample Prep Kit. Gene expression levels were qualified using transcripts per million (TPM) as the metric. Differentially expressed genes (DGEs) were identified using DESeq2 (http://bioconductor.org/packages/stats/bioc/DESeq2/) with the criteria of false discovery rate (FDR) < 0.05 and |log2 fold change| ≧ 1; genes meeting both criteria were considered DEGs. The raw RNA‐seq data have been deposited in the NCBI Sequence Read Archive under Accession Number PRJNA987992.

#### 2.4.7. Untargeted Metabolite Analysis

Untargeted metabolite profiling of intestine samples was performed using an ultra‐high‐performance liquid chromatography‐tandem mass spectrometry (UHPLC–MS/MS) system (Vanquish UHPLC, Thermo Fisher, Germany) coupled with an Orbitrap Q Extractive HF‐X mass spectrometer (Thermo Fisher, Germany) at Gene Denovo Co., Ltd. (Guangzhou, China). The raw UHPLC‐MS/MS data were processed with XCMS for peak alignment, detection, and quantitation. Peak intensities were normalized to the total ion current of the first samples to ensure comparability across samples. Metabolite identification was achieved by matching accurate masses (mass deviation ≦10 ppm) and adduct ion forms against a high‐quality MS^2^ spectral database, providing reliable qualitative and relative quantitative information. Statistical analyses were conducted using R (Version R‐3.4.3), Python (Version 2.7.6), and CentOS (Version 6.6). For nonnormally distributed data, normalization was performed according to the formula: sample raw quantitation value / (sum of sample metabolite quantitation value / sum of first‐sample metabolite quantitation value), yielding relative peak areas. Differential metabolites were identified using an unpaired Student’s *t*‐test, and those with a *p* < 0.05 were considered statistically significant.

### 2.5. Statistical Analysis

Data were tested for normality and homogeneity of variances using the Shapiro–Wilk and Levene’s tests, respectively. One‐way analysis of variance (ANOVA) followed by Tukey’s post hoc test was performed to evaluate statistical differences among groups using GraphPad Prism 8 (GraphPad Software, La Jolla, CA, USA). Differences were considered statistically significant at *p*  < 0.05 ( ^∗^) and *p*  < 0.01 ( ^∗∗^). Data were presented as the mean ± standard error of the mean (SEM).

## 3. Results

### 3.1. Antibacterial Activity of Yeast Polysaccharides Against Vibrio Spp

**Figure 1:**
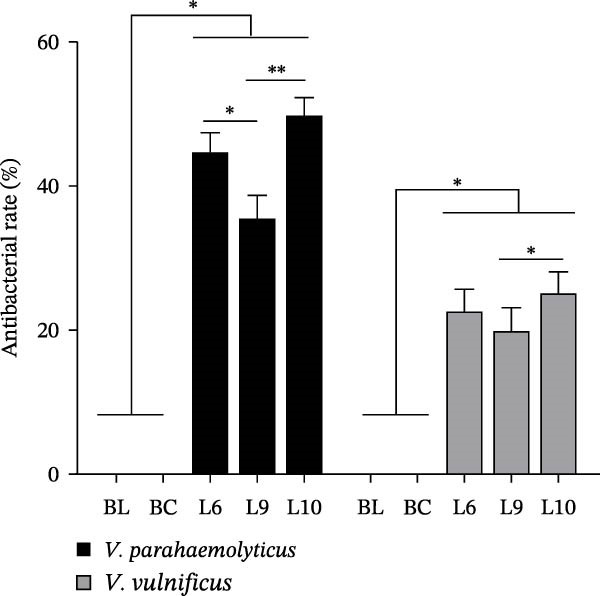
Antibacterial effects of different yeast polysaccharide sources against *Vibrio* spp. Values are presented as mean ± SEM (*n* = 9). BL (blank control): sterile PBS only; BC (bacterial control): *Vibrio* + PBS + distilled water. Asterisks denote significant differences among treatments ( ^∗^ *p*  < 0.05;  ^∗∗^ *p*  < 0.01).

The antibacterial activities of yeast polysaccharides derived from L6, L9, and L10 strains are presented in Table 3 and Figure 1. Colonies detected in the blank control (BL) were identified as environmental contaminants, accounting for below 0.01% of those in the BC, thereby confirming the absence of experimental contamination. Compared with BC, YPS treatments with L6, L9, and L10 significantly reduced *Vibrio* colony counts (*p* < 0.05), and three YPS preparations exhibited significant antibacterial activity against both *Vibrio parahaemolyticus* (VP32) and *Vibrio vulnificus* (VP44) (*p* < 0.05). Inhibition rates were ~45%, 36%, and 50% against *V. parahaemolyticus* for L6, L9, and L10, respectively, with L10 showing the strongest antibacterial effect (*p* < 0.01 vs. L9). In contrast, inhibition of *V. vulnificus* was relatively lower, ranging from 20% to 26%, with L10 again exhibiting the highest activity among the three preparations (*p* < 0.05 vs. L9).

**Table 3:** Colony counts (CFU/mL) of *Vibrio* spp. in response to supplementation with different yeast polysaccharide sources.

Items	BL	BC	L6	L9	L10
*V. parahaemolyticus* (VP32)	63 ± 4	98 ± 4.8 × 10^4a^	54 ± 3.5 × 10^4cd^	63 ± 3.8 × 10^4b^	49 ± 3.2 × 10^4d^
*V. vulnificus* (VP44)	72 ± 3	95 ± 4.2 × 10^4a^	74 ± 3.2 × 10^4bc^	76 ± 3.3 × 10^4b^	71 ± 3.1 × 10^4c^

*Note*: BL (blank control): sterile PBS only; BC (bacterial control): *Vibrio* + PBS + distilled water. Data are presented as mean ± SEM (*n* = 9). Different superscript letters indicate significant differences among groups (*p*  < 0.05, one‐way ANOVA with Duncan’s test). The superscript letters a, b, c, d denote statistical significance: different letters indicate significant differences among groups at *p* < 0.05 (one‐way ANOVA with Duncan’s test), while values sharing the same letter are not significantly different.

### 3.2. Growth Performance and Feed Utilization

The growth performance and feed utilization of red tilapia are detailed in Table 4. Survival rates exceeded 98% across all treatments, indicating that dietary yeast polysaccharides were safe and had no adverse effects on fish survival. All yeast polysaccharide‐supplemented diets enhanced growth performance compared with the nonsupplemented control group. Notably, L10 produced the highest final body weight (FW, 855.89 ± 6.79 g/fish), weight gain rate (WGR, 4180 ± 41.3%), and specific growth rate (SGR, 3.54 ± 0.01%/d), along with the lowest feed conversation ratio (FCR, 0.71 ± 0.01) (*p* < 0.05). S, L6, and L9 also resulted in significant increases in FW, WGR, and SGR, accompanied by lower FCR values (0.72–0.74), suggesting improved feed efficiency. In contrast, the Y group showed growth performance similar to the control in FW, WGR, and SGR but a slightly lower FCR, indicating marginally better feed utilization.

**Table 4:** Effects of different sources of yeast polysaccharides supplementation on the growth and feed utilization in red tilapia.

Items	C	Y	S	L6	L9	L10
IW^1^ (g/fish)	20.11 ± 0.22	20.25 ± 0.08	20.00 ± 0.14	20.00 ± 0.19	19.92 ± 0.16	20.00 ± 0.14
FW^2^ (g/fish)	805.50 ± 13.25^a^	825.00 ± 9.50^ab^	829.38 ± 10.24^abc^	834.13 ± 6.73^bc^	826.13 ± 5.03^ab^	855.89 ± 6.79^c^
WGR^3^ (%)	3904 ± 36.3^a^	3974 ± 54.3^a^	4048 ± 69.6^ab^	4072 ± 58.7^ab^	4049 ± 38.5^ab^	4180 ± 41.3^b^
SGR^4^ (%/d)	3.48 ± 0.01^a^	3.50 ± 0.01^a^	3.51 ± 0.02^ab^	3.52 ± 0.01^ab^	3.51 ± 0.01^ab^	3.54 ± 0.01^b^
FCR^5^	0.78 ± 0.01^c^	0.73 ± 0.01^ab^	0.74 ± 0.02^b^	0.72 ± 0.01^ab^	0.74 ± 0.00^b^	0.71 ± 0.01^a^
SR^6^ (%)	98.67 ± 1.33	99.50 ± 0.50	99.00 ± 1.00	98.00 ± 0.82	99.50 ± 0.50	98.00 ± 1.15

*Note*: Data are presented as Mean ± SEM (*n* = 4), with different letters indicating differences within the groups (*p* < 0.05). The superscript letters a, b, c indicate significant differences among treatments at *p* < 0.05 (one‐way ANOVA followed by Duncan’s multiple range test). Values with different superscript letters are significantly different, while those sharing the same letter are not.

^1^Initial average body weight.

^2^Final average body weight.

^3^Weight gain rate (WGR) (%) = 100 × (FW (g) − IW (g)) / IW (g).

^4^Specific growth rate (SGR) (%/d) = 100 × ((Ln (FW (g)) − Ln (IW (g))) / days).

^5^Feed conversion ratio (FCR) = dry feed fed (g) / wet weight gain (g).

^6^Survival rate (SR) (%) = 100 × (final number of fish/initial number of fish).

### 3.3. Serum biochemical parameters

**Figure 2:**
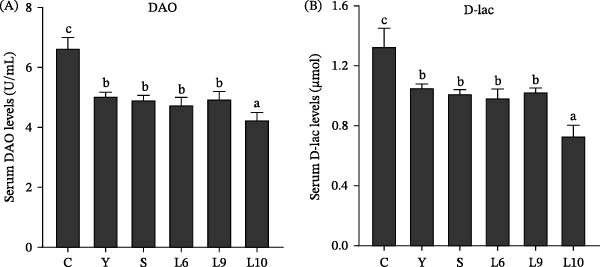
Effects of dietary yeast polysaccharide supplementation on serum intestinal barrier‐related parameters in red tilapia. Values are expressed as mean ± SEM (*n* = 4) (A) shows the effects of dietary yeast polysaccharide supplementation on serum diamine oxidase (DAO) levels, and subpanel (B) shows the effects on serum D‐lactic acid (D‐lac) levels. Different superscript letters indicate significant differences among treatments (*p* < 0.05). Measured indices: DAO, diamine oxidase; D‐lac, D‐lactic acid.

As presented in Table 5, serum biochemical indices were significantly influenced by dietary yeast polysaccharide supplementation (*p* < 0.05). The control group exhibited the highest levels of TC, TG, Glu, and MDA, along with the lowest activities of AKP, T‐AOC, LYZ, and complement components C3 and C4 (*p* < 0.05). In contrast, all yeast polysaccharide‐supplemented groups exhibited reduced TC, TG, Glu, and MDA, accompanied by significantly elevated AKP, T‐AOC, LYZ, C3, and C4 compared with the control (*p* < 0.05). Among treatments, L10 yields the most favorable biochemical profile, with the lowest TC, TG, Glu, and MDA levels and the highest AKP, T‐AOC, LYZ, C3, and C4 activities. Furthermore, serum biomarkers associated with intestinal barrier function, including D‐lactate (D‐lac) and diamine oxidase (DAO) (Figure 2), were significantly decreased in all yeast polysaccharide groups relative to the control (*p* < 0.05), with L10 showing the lowest values. No significant differences were observed among the other supplemented groups (*p* > 0.05).

**Table 5:** Effects of yeast culture on serum biochemical parameters of red tilapia.

Items	C	Y	S	L6	L9	L10
TC (mmol/L)	4.81 ± 0.09^c^	4.56 ± 0.33^bc^	4.48 ± 0.15^ab^	4.47 ± 0.27^ab^	4.51 ± 0.06^bc^	4.17 ± 0.13^a^
TG (mmol/L)	2.74 ± 0.34^b^	2.41 ± 0.22^a^	2.37 ± 0.13^a^	2.22 ± 0.04^a^	2.39 ± 0.10^a^	2.10 ± 0.18^a^
Glu (mmol/L)	5.50 ± 0.40^b^	4.96 ± 0.14^a^	4.88 ± 0.20^a^	4.83 ± 0.12^a^	4.95 ± 0.26^a^	4.74 ± 0.22^a^
AKP (U/L)	2.20 ± 0.14^a^	2.57 ± 0.10^b^	2.69 ± 0.25^b^	2.82 ± 0.18^b^	2.66 ± 0.13^b^	3.11 ± 0.12^c^
MDA (nmol/mL)	4.46 ± 0.09^c^	4.45 ± 0.13^b^	4.38 ± 0.05^b^	4.35 ± 0.10^b^	4.41 ± 0.13^b^	3.98 ± 0.05^a^
T‐AOC (mM)	5.42 ± 0.14^a^	6.45 ± 0.33^b^	7.33 ± 0.18^c^	7.39 ± 0.23^c^	6.75 ± 0.25^b^	8.39 ± 0.25^d^
LYZ (U/mL)	87.53 ± 4.15^a^	106.15 ± 4.63^b^	126.65 ± 4.87^c^	130.60 ± 5.60^c^	121.38 ± 21.06^c^	149.25 ± 3.48^d^
C3 (μg/mL)	83.91 ± 3.54^a^	105.67 ± 4.24^b^	111.85 ± 3.06^c^	119.48 ± 3.18^d^	110.02 ± 4.09^bc^	133.80 ± 4.81^e^
C4 (ug/mL)	22.20 ± 1.29^a^	33.26 ± 0.48^b^	34.37 ± 1.49^bc^	35.06 ± 0.60^c^	33.77 ± 0.97^bc^	38.24 ± 0.56^d^

*Note*: Data are presented as Mean ± SEM (*n* = 4), with different letters indicating differences within the groups (*p* < 0.05). Measured indices include TC, total cholesterol; TG, triglyceride; Glu, glucose; MDA, malondialdehyde; AKP, alkaline phosphatase; T‐AOC, total antioxidant capacity; LYZ, lysozyme; and complement components C3 and C4. The superscript letters a, b, c represent significant differences between treatments at *p* < 0.05 (one‐way ANOVA followed by Duncan’s multiple range test). Values with different superscript letters indicate significant differences, while values sharing the same letter show no significant difference.

### 3.4. Intestinal Cytokine Secretion

**Figure 3:**
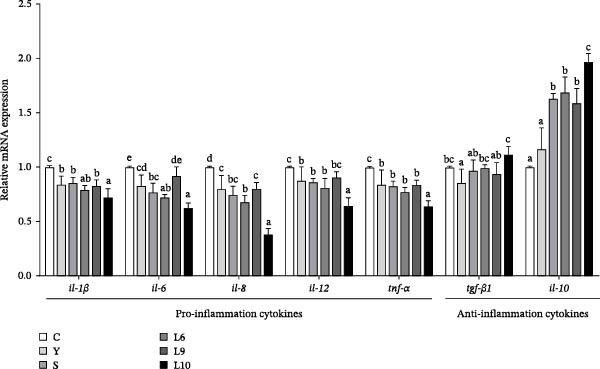
Effects of dietary yeast polysaccharide supplementation on intestinal cytokine secretion in red tilapia. Pro‐inflammatory cytokines include *il-1β*, interleukin 1β; *il-12*, interleukin 12; *il-8*, interleukin 8; *il-6*, interleukin 6; and *tnf-α*, tumor necrosis factor‐α. Anti‐inflammatory cytokines include *tgf-β1*, transforming growth factor‐β1 and *il-10*, interleukin 10. Values are expressed as mean ± SEM (*n* = 4). Different superscript letters indicate significant differences among treatment groups (*p* < 0.05).

The intestinal expression levels of proinflammatory cytokines (*il-1β*, *il-6*, *il-8*, *il-12*, and *tnf-α*) and anti‐inflammatory cytokines (*tgf-β1* and *il-10*) are illustrated in Figure 3. Significant differences were observed among treatments (*p* < 0.05). Compared with the control group, dietary yeast polysaccharide supplementation significantly reduced proinflammatory cytokine levels while upregulating *tgf-β1* and *il-10* expression (*p* < 0.05). Among the treatments, L10 exhibited the strongest immunomodulatory effect, characterized by the lowest expression of *il-1β*, *il-6*, *il-8*, *il-12*, and *tnf-α*, together with the highest expression of *tgf-β1* and *il-10* (*p* < 0.05).

### 3.5. Intestinal Morphology

**Figure 4:**
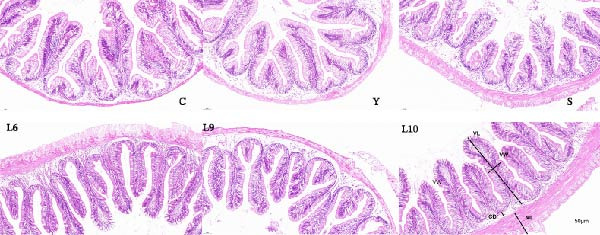
Representative histological sections of the mid‐intestine of red tilapia fed different diets (H&E staining). C, control; Y, a commercial yeast β‐glucan product (≥80% β‐glucan); S, a commercial mannan oligosaccharide product (≥70% MOS); L6, L9, and L10, yeast polysaccharides isolated from strains L6, L9, and L10. Abbreviations: CD, crypt depth; ML, muscularis thickness; VL, villus length; VW, villus width. Scale bar = 50 μm.

**Figure 5:**
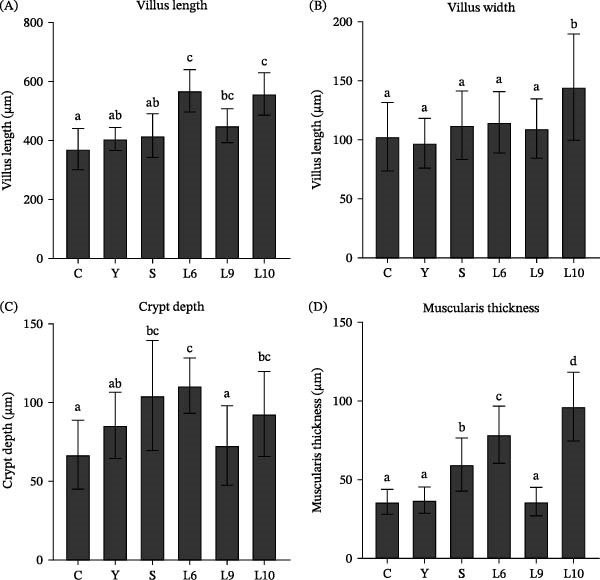
Effects of dietary yeast polysaccharide supplementation on intestinal morphology of red tilapia. (A) Villus length, (B) villus width, (C) crypt depth, and (D) muscularis thickness. Values are presented as means ± SEM (*n* = 15). Different superscript letters indicate significant differences among treatments (*p* < 0.05).

Histological examination of the hindgut revealed clear differences among treatments (Figure 4). In the control (C) and Y groups, villi were relatively short and loosely arranged, with a thinner muscular layer, while the S group showed only slight improvements. Fish fed L6, L9, and particularly L10 exhibited elongated, densely packed villi, deeper crypts, and thicker muscular layers. Compared with the control, quantitative analysis in Figure 5 showed significantly increased villus length in the L6 and L10 groups and evaluated villus width only in L10 (*p* < 0.05). Crypt depth was greater in the S, L6, and L10 groups than in the control (*p* < 0.05), while muscularis thickness was most pronounced in L6 and L10, with L10 exhibiting the greatest value among all treatments (*p* < 0.05).

### 3.6. Taxonomic Composition of the Intestinal Microbiota

**Figure 6:**
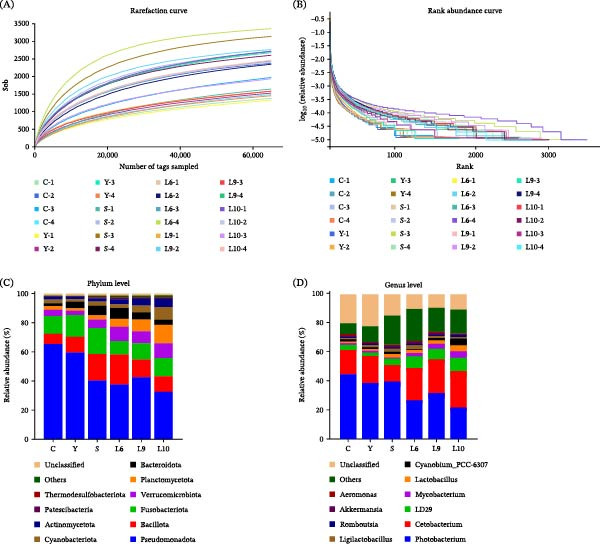
Effect of dietary yeast polysaccharide supplementation on the intestinal microbiota in red tilapia. (A) Rarefaction curve; (B) Rank abundance curve; (C) Relative abundance of the top 10 bacterial phyla; (D) Relative abundance of the top 10 bacterial genera.

A total of 2,084,829 high‐quality reads were obtained, averaging 86,867 reads per sample. Rarefaction analysis clustered these reads into 2378 operational taxonomic units (OTUs). The Good’s coverage values (99.24% to 99.38%; Table 6) indicated that the sequencing depth was sufficient to represent the intestinal microbial community. Both rarefaction and rank‐abundance curves approached saturation (Figure 6A,B), further confirming adequate sequencing depth and coverage of microbial diversity. Significant differences were detected among groups in OUT numbers and diversity indices, including Shannon and Simpson values (*p* < 0.05; Table 6). Although ACE and Chao1 indices showed no significant differences among treatments (*p* > 0.05), the S, L6, L9 and L10 groups generally exhibited higher species richness compared with the control and Y groups. Among them, L6 displayed the highest ACE and Chao1 values, while L10 also maintained elevated richness. For diversity indices, Shannon and Simpson values were significantly higher in the L6, L9, and L10 groups than in the control (*p* < 0.05). The Y and S groups also showed an upward trend in these indices, though not always statistically significant (*p* > 0.05).

**Table 6:** Effects of different yeast polysaccharide sources on intestinal microbiota α‐diversity of red tilapia.

Items	Richness estimates			Diversity estimates		Coverage (%)
	OTUs	ACE	Chao1	Simpson	Shannon	
C	1732 ± 237^a^	2434 ± 262	2401 ± 265	0.862 ± 0.012^a^	4.75 ± 0.23^a^	99.24 ± 0.03
Y	1901 ± 354^ab^	2470 ± 290	2437 ± 332	0.892 ± 0.024^ab^	5.23 ± 0.67^ab^	99.32 ± 0.03
S	2471 ± 350^ab^	2909 ± 213	2917 ± 272	0.915 ± 0.033^ab^	6.02 ± 0.81^ab^	99.38 ± 0.07
L6	2778 ± 254^b^	3217 ± 164	3239 ± 195	0.939 ± 0.011^b^	6.86 ± 0.68^b^	99.27 ± 0.14
L9	2567 ± 397^ab^	3013 ± 280	3050 ± 307	0.946 ± 0.011^b^	6.86 ± 0.55^b^	99.38 ± 0.03
L10	2781 ± 115^b^	3175 ± 103	3218 ± 104	0.955 ± 0.008^b^	7.00 ± 0.27^b^	99.34 ± 0.05

*Note*: Data are presented as Mean ± SEM (*n* = 4), with different letters indicating differences within the groups (*p* < 0.05). The superscript letters a, b indicate significant differences among treatments at *p* < 0.05 (one‐way ANOVA followed by Duncan’s multiple range test). Values with different superscript letters are significantly different, while those sharing the same letter are not.

All groups were dominated by *Pseudomonadota* (formerly *Proteobacteria*), *Bacillota* (formerly *Firmicutes*), and *Fusobacteriota*, followed by *Verrucomicrobiota*, *Planctomycetota*, *Bacteroidota*, and *Cyanobacteriota* at the phylum level (Figure 6C). Particularly in the L10 group, the relative abundance of *Pseudomonadota* was markedly reduced compared with the control, whereas *Verrucomicrobiota* and *Planctomycetota* increased substantially. At the genus level (Figure 6D), *Photobacterium* and *Cetobacterium* remained the most abundant genus in all groups, followed by *LD29*, *Mycobacterium* and *Lactobacillu*s. When fish were fed with yeast polysaccharides derived from L6, L9, and L10, the abundance of *Photobacterium* was markedly reduced while *Cetobacterium* increased; meanwhile, a higher abundance of potentially beneficial bacteria, including *LD29*, lactic acid bacteria (*Lactobacillus* and *Ligilactobacillus*), and *Akkermansia*, was observed.

### 3.7. Transcriptomic and Metabolomic Alterations of the Intestine

**Figure 7:**
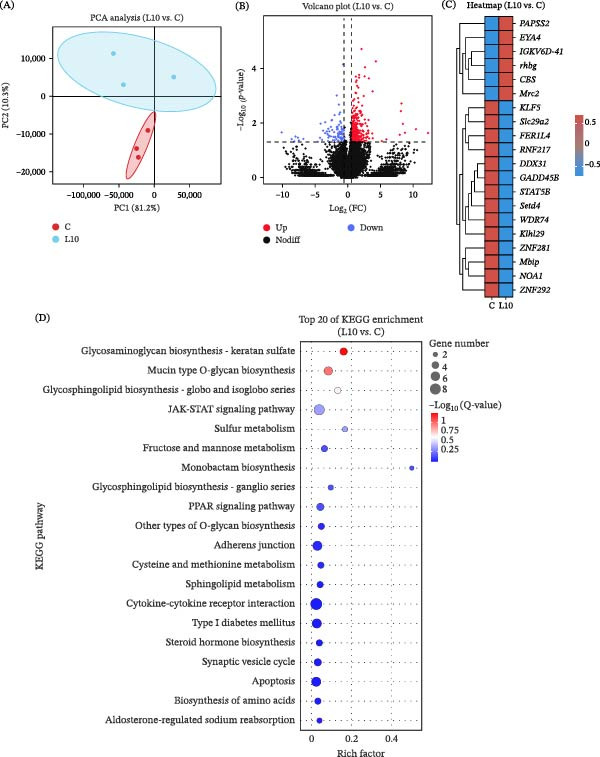
Effects of yeast polysaccharide derived from strain L10 on the transcriptomic features of red tilapia intestine. (A) PCA score plot for the transcriptomic profiles between C and L10 groups; (B) Volcano plot of differentially expressed genes (DEGs); (C) Heatmap of representative DEGs; (D) Bubble diagram of KEGG enrichment analysis of DEGs. C, control without yeast polysaccharide; L10, 1 g/kg yeast polysaccharide derived from strain L10.

Transcriptomic and metabolomic analyses were performed to gain deeper insight into the mechanisms underlying the effects of dietary L10 supplementation in red tilapia. Principal component analysis (PCA) revealed a clear separation between the control (C) and L10 groups, indicating substantial transcriptional reprograming induced by yeast polysaccharides (Figure 7A). Volcano plot analysis identified numerous DEGs, with the majority being upregulated in the L10 group (Figure 7B). Heatmap clustering further highlighted the pronounced upregulation of key immune‐ and metabolic homeostasis‐related genes, such as *PAPSS2* (3^′^‐phosphoadenosine‐5^′^‐phosphosulfate synthase 2), *EYA4* (eye absent homolog 4), *rhbg* (Rhesus blood group‐associated glycoprotein b), and *CBS* (Cystathionine β‐synthase) in the L10 group (Figure 7C). KEGG pathway enrichment analysis (Figure 7D) showed that these DEGs were primarily involved in glycosaminoglycan and glycosphingolipid biosynthesis, JAK‐STAT signaling, cytokine–cytokine receptor interaction, and the PPAR signaling pathway.

**Figure 8:**
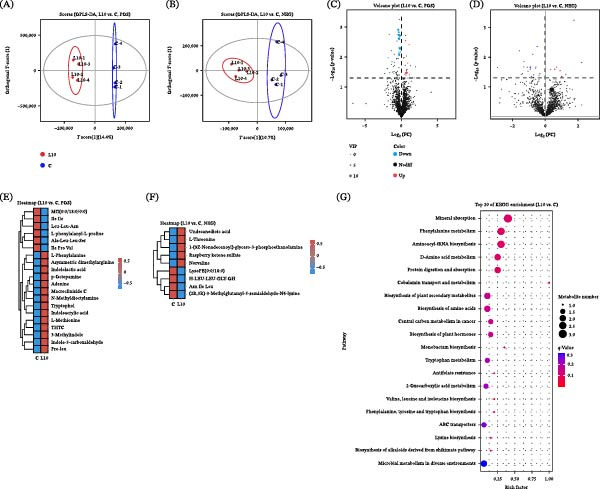
Effects of yeast polysaccharide derived from strain L10 on the metabolomic features in red tilapia intestine. (A, B) OPLS‐DA score plot for the metabolomic profiles between C and L10 under positive ion mode (POS) and negative ion mode (NEG); (C, D) Volcano plot of significantly altered metabolites under POS and NEG; (E, F) Heatmap of representative metabolites in the C and L10 group under POS and NEG; (G) KEGG pathway enrichment analysis of differential metabolites. C, control without yeast polysaccharide; L10, 1 g/kg yeast polysaccharide derived from strain L10.

Metabolomic profiling also revealed distinct reprograming between the control (C) and L10 groups, as demonstrated by orthogonal projections to latent structures – discriminant analysis (OPLS‐DA) (Figure 8A,B). Volcano plot (Figure 8C,D) and heatmap analyses (Figure 8E,F) confirmed substantial shifts in metabolite accumulation. Notably, several essential amino acids, including L‐phenylalanine, L‐methionine, and L‐threonine, were significantly upregulated in the L10 group (Table 7), with KEGG enrichment analysis indicating involvement in amino acid metabolism, aminoacyl‐tRNA biosynthesis, protein digestion and absorption, and secondary metabolite biosynthesis (Figure 8G). Indole derivatives such as tryptophol and 3‐methylindole, as well as adenine and p‐octopamine, were markedly increased when fish were fed yeast polysaccharide from strain L10, while (2R,3R)‐3‐methylglutamyl‐5‐semialdehyde‐N6‐lysine, a metabolite involved in lysine biosynthesis, was downregulated (Table 7).

**Table 7:** Annotation results of differentially expressed metabolite KEGG in the control and L10 group (C vs. L10, *n* = 4).

Metabolites	Up or down	Log_2_FC	VIP	KEGG pathway annotation
L‐phenylalanine	Up	0.20	10.86	Mineral absorption (ko04978); phenylalanine metabolism (ko00360); aminoacyl‐tRNA biosynthesis (ko00970); D‐amino acid (ko00470); protein digestion and absorption (ko04974); biosynthesis of plant second metabolites (ko01060); biosynthesis of amino acids (ko01230); central carbon metabolism in cancer (ko05230); biosynthesis of plant hormones (ko01070); 2‐oxocarboxylic acid metabolism (ko01210); phenylalanine, tyrosine, and tryptophan biosynthesis (ko00400); ABC transporters (ko02010); biosynthesis of alkaloids derived from shikimate pathway (ko01063); biosynthesis of phenylpropanoids (ko01061); biosynthesis of alkaloids derived from ornithine, lysine, and nicotinic acid (ko01064); biosynthesis of secondary metabolites (ko01110); metabolic pathways (ko01100)
L‐methionine	Up	0.42	8.18	Mineral absorption (ko04978); aminoacyl‐tRNA biosynthesis (ko00970); D‐amino acid (ko00470); protein digestion and absorption (ko04974); cobalamin transport and metabolism (ko04980); biosynthesis of plant second metabolites (ko01060); biosynthesis of amino acids (ko01230); central carbon metabolism in cancer (ko05230); biosynthesis of plant hormones (ko01070); altifolate resistance (ko01523); 2‐oxocarboxylic acid metabolism (ko01210); cysteine and methionine metabolism (ko00270); biosynthesis of secondary metabolites (ko01110); metabolic pathways (ko01100); biosynthesis of cofactors (ko01240)
p‐Octopamine	Up	0.30	6.29	Neuroactive ligand–receptor interaction (ko04080)
Tryptophol	Up	0.33	4.37	Tryptophan metabolism (ko00380)
3‐methylindole	Up	0.32	1.76	Tryptophan metabolism (ko00380)
Adenine	Up	1.06	1.17	Biosynthesis of secondary metabolites (ko01110); purine metabolism (ko00230); nucleotide metabolism (ko01232); metabolic pathways (ko01100)
L‐threonine	Up	0.49	2.17	Mineral absorption (ko04978); aminoacyl‐tRNA biosynthesis (ko00970); D‐amino acid (ko00470); protein digestion and absorption (ko04974); biosynthesis of plant second metabolites (ko01060); biosynthesis of amino acids (ko01230); monobactam biosynthesis (ko00261); valine, leucine, and isoleucine biosynthesis (ko00290); ABC transporters (ko02010); microbial metabolism in diverse environments (ko01120); porphyrin metabolism (ko008600); glycine, serine, and threonine metabolism (ko00260); biosynthesis of secondary metabolites (ko01110); metabolic pathways (ko01100)
(2R,3R)‐3‐methylglutamyl‐5‐semialdehyde‐N6‐lysine	Down	−0.32	1.18	Lysine biosynthesis (ko00300); microbial metabolism in diverse environments (ko01120); metabolic pathways (ko01100)

## 4. Discussion

*Vibrio* spp. secretes multiple hemolytic toxins that compromise epithelial cell and hemocyte membrane integrity, resulting in extensive cellular damage and accelerated host mortality [29, 30]. Therefore, it is urgent to develop sustainable, nonantibiotic alternatives to mitigate *Vibrio*‐associated disease risks in aquaculture systems. The present study demonstrated that yeast polysaccharides (YPS) derived from *Saccharomyces cerevisiae* exhibited clear antibacterial activity against *V. parahaemolyticus* and *V. vulnificus*, with notable strain‐dependent differences. YPS from strain L10 showed stronger inhibitory activity than L6 and L9, suggesting that structural or compositional variations among polysaccharides significantly influence their antimicrobial potential. It is known that differences in β‐glucans content, molecular weight, and linkage pattern were found to modulate the immunostimulatory and pathogen‐binding capacity of yeast cell wall polysaccharides [31, 32]. Moreover, the beading–milling method employed here likely enhanced the exposure of polysaccharide chains and disrupted cell wall crosslinking, improving solubility and bioavailability. This is consistent with previous findings that industrial or mechanical treatments can induce cell wall remodeling, altering the structural integrity and biological activity of YPS [33, 34]. In addition, the yeast cell wall is inherently dynamic and responsive to environmental stressors, undergoing continuous remodeling to maintain functional integrity [35]. Thus, processing‐induced structural modifications could partly explain the enhanced antibacterial properties of specific YPS preparations observed in this study. Notably, the antibacterial effects of YPS were more pronounced against *V. parahaemolyticus* than against *V. vulnificus*, suggesting differences in cell surface composition or adhesion mechanism between species. This might be linked to variations in host recognition pathways mediated by pattern recognition receptors such as dendritic cell‐associated C‐type lectin‐1 (Dectin‐1), which binds β‐glucan motifs and mediates downstream immune activation [36]. Therefore, the superior inhibitory activity of L10 may reflect a higher proportion of biologically active β‐(1 → 3)‐glucan or mannan residues that facilitate recognition or binding to bacterial surface components, implying that polysaccharide structure‐function relationships play a decisive role in modulating host‐pathogen interactions.

The subsequent in vivo evaluation of L6, L9, and L10 provides a crucial step toward elucidating the mechanistic basis of YPS‐mediated health promotion in red tilapia. Similar to various aquaculture species [17, 19, 20, 37–47], in the present study, dietary supplementation with YPS, particularly with L10, markedly enhanced growth performance and feed efficiency. However, most previously reported studies have been limited to short‐term trials, leaving the long‐term effects of YPS supplementation under seawater conditions—where environmental stressors such as salinity fluctuations, osmotic challenges, and oxidative stress are more pronounced—largely unexplored. In the present study, we report for the first time throughout the entire production cycle (~16 weeks) of red tilapia, from juveniles (~20 g) to market size (~800 g) under seawater conditions (20–25 ppm). These long‐term physiological benefits in our present study highlight the practical value of yeast polysaccharides as functional feed additives, supporting their application in sustainable marine aquaculture systems by promoting fish health, reducing reliance on chemotherapeutic agents, and improving production efficiency over extended culture periods. Compared with commercial β‐glucan (Y) and MOS (S) products, L10 supplementation achieved superior weight gain and feed conversion, which are likely attributable to the distinctive structural features of L10, including enhanced solubility and favorable β‐glucan and mannose configurations. Previous studies have shown that glycosidic linkage types, monosaccharide composition, molecular weight, and chain conformation of β‐glucans could influence their physicochemical characteristics and biological activities [48, 49].

Consistent with previous observations in tilapia and other teleosts [10, 50, 51], YPS have been shown to promote health and performance. In our present study, fish receiving L10 exhibited markedly reduced serum levels of MDA, TC, TG, and Glu, along with increased activities of AKP, T‐AOC, LYZ, and complement components C3 and C4. These biochemical changes collectively reflect an improved oxidative balance and potentiation of both innate and humoral immunity. Furthermore, downregulation of proinflammatory mediators (*il-1β*, *il-6*, *il-8*, *il-12*, and *tnf-α*) while upregulation of anti‐inflammatory factors (*tgf-β1* and *il-10*) by L10 indicated a fine‐tuning of immune homeostasis, which may alleviate physiological stress, optimize nutrient utilization, and maintain immune balance—thereby fostering an internal environment conducive to efficient growth.

Maintaining the structural and functional integrity in the intestine is crucial for optimal digestion and nutrient absorption in fish [52, 53]. In the present study, increased villus height, crypt depth, and muscularis thickness were observed in the L10 group, indicating the expansion of the absorptive surface, facilitating nutrient uptake and supporting improved growth performance [54, 55]; meanwhile, the lowest serum D‐lac and DAO levels when fish were fed with L10 further confirmed the reinforcement of intestinal barrier integrity. Similar effects have been observed in other aquatic species [38, 40, 56, 57], suggesting that YPS enhance gut health by simultaneously supporting the intestinal structure and barrier function, which in turn promotes overall nutrient utilization and growth.

Growing evidence showed the gut microbiota plays an important role in the health of the host [53, 58], and polysaccharides fermented by the gut microbiota could exert various beneficial effects, including anti‐inflammation, barrier protection, and immune regulation on the host intestine through a microbiota‐dependent or ‐independent mechanism [59]. Beyond the above improvements, in our present study, yeast polysaccharide L10 supplementation enhanced both diversity and functional stability of intestinal microbiota in red tilapia, characterized by concurrent enrichment of *Bacteroidota*, *Verrucomicrobiota*, and *Planctomycetota*, along with the pronounced reduction in *Pseudomonadota* at the phylum level. It is known that phylum *Bacteroidota* plays a vital role in maintaining intestinal immune homeostasis and supporting host health [60]. In contrast, *Pseudomonadota*—the largest bacterial phylum encompassing pathogenic genera such as *Escherichia coli*, *Salmonella*, *Vibrio cholerae*, and *Helicobacter pylori*—is often considered a microbial indicator of disease risk [61]. The phylum *Verrucomicrobiota* includes beneficial species such as *Akkermansia muciniphila*, a mucin‐degrading bacterium essential for preserving intestinal barrier integrity [62]. Similarly, *Planctomycetota* contribute to the degradation of complex glycoproteins and fungal polysaccharides and play a role in carbon and nitrogen recycling [63]. The changes in these microbial communities suggest that L10 yeast polysaccharides remodel the intestinal microbiota toward a more balanced, health‐associated configuration, thereby suppressing pathogenic taxa and enhancing host resilience. In addition, the genus *Photobacterium* includes pathogenic species such as *P. damselae* subsp. *damselae*, which causes wound infections and hemorrhagic septicemia in fish [64]. In contrast, *Cetobacterium* produces vitamin B_12_ that supports the overall nutritional status of fish and generates antimicrobial metabolites that enhance immune function [65]. The observed decrease in *Photobacterium* and increase in *Cetobacterium* in the L10 group indicates that L10‐derived yeast polysaccharides help suppress opportunistic pathogens and stimulate beneficial microbial metabolism and thereby reduce disease risk while promoting a healthier intestinal ecosystem. Lactic acid bacteria, including *Lactobacillus* and *Ligilactobacillus*, support intestinal barrier integrity and inhibit pathogens via ecological competition and bacteriocin production, thereby enhancing nutrient digestion, feed intake, and growth performance [66]. Furthermore, L10 yeast polysaccharides may serve as fermentable substrates, enriching microbial populations such as *LD29*, *Lachnospiraceae*, and *Alistipes*, which are potential producers of propionate and butyrate, linking polysaccharide supplementation to beneficial metabolic outcomes [67].

Transcriptomic and metabolomic analyses further reveal the potential mechanisms of improvements by L10 yeast polysaccharide in regulating the antioxidant capacity, immunity, metabolism, and overall homeostasis of red tilapia. At the transcriptomic level, L10 supplementation primarily affected genes related to sulfur metabolism and redox regulation. The significant upregulation of cystathionine β‐synthase (*CBS*), a key enzyme in the transsulfuration pathway, suggests an increased capacity for cysteine biosynthesis and subsequent glutathione (GSH) production [68]. Given the central role of GSH in maintaining cellular redox balance and supporting antioxidant and immune functions, enhanced CBS‐mediated transsulfuration provides a plausible nutritional explanation for the improved antioxidant status and reduced inflammatory responses observed in red tilapia fed the L10 yeast polysaccharide [69]. In parallel, L10 supplementation markedly increased the expression of *PAPSS2*, which catalyzes the formation of 3^′^‐phosphoadenosine‐5^′^‐phosphosulfate (PAPS), the universal sulfate donor for the sulfation reaction [70]. Sulfation is a critical phase II metabolic process involved in the regulation and detoxification of both endogenous compounds and microbial‐derived metabolites [71]. The upregulation of PAPSS2 therefore indicates an enhanced sulfation potential in intestinal tissues, contributing to improved metabolic homeostasis and adaptive capacity in response to dietary intervention. Furthermore, the elevated expression of *EYA4*, a multifunctional regulator involved in several stress‐ and growth‐related signaling pathways, suggests a supportive role in cellular maintenance and tissue homeostasis [72]. The concurrent upregulation of *rhbg* is associated with ammonia handling and metabolic stability under culture‐related stress conditions [73]. Collectively, these transcriptomic changes indicate that L10 yeast polysaccharide modulates sulfur‐related metabolic pathways and associated adaptive responses, thereby supporting redox balance and intestinal metabolic resilience in red tilapia.

Consistent with these transcriptomic alterations, metabolomic profiling revealed significant changes in indole‐derived metabolites, including tryptophol, 3‐methylindole, indolelactic acid, and indoleacrylic acid. These compounds are primarily produced by gut microbiota through tryptophan metabolism and are recognized as endogenous ligands of the aryl hydrocarbon receptor (AhR) [74, 75]. Previous studies have demonstrated that indole‐3‐acetic acid (IAA), another microbial tryptophan metabolite, enhances intestinal mucin sulfation via the AHR‐PAPSS2‐SLC35B3 axis, thereby contributing to the maintenance of intestinal homeostasis [76]. In this context, the increased abundance of indole derivatives observed in the present study suggests that L10 yeast polysaccharide may promote microbial tryptophan metabolism toward AhR activation, which is consistent with the concurrent upregulation of sulfur metabolism‐ and sulfation‐related genes identified at the transcriptomic level. Although the direct involvement of the AhR axis in mediating the protective effects of L10 yeast polysaccharides through sulfur metabolism and sulfation pathways requires further experimental validation, the coordinated changes in both indole metabolites and key sulfation enzymes provide supportive evidence for a diet‐microbiota‐host metabolic interaction. Elucidating this regulatory axis would offer valuable mechanistic insight for the rational development of targeted functional feed additives in marine aquaculture. In addition to tryptophan metabolism, L10 supplementation was associated with reduced levels of intermediates involved in lysine biosynthesis, which may reflect a redistribution of amino acid metabolic flux, favoring protein synthesis and immune‐related processes rather than de novo amino acid production. Meanwhile, the accumulation of metabolites such as adenine and p‐octopamine indicates modulation of nucleotide metabolism and stress‐related signaling pathways, which are known to participate in the energy balance, cellular adaptation, and stress responsiveness [77, 78]. Collectively, these metabolomic shifts suggest that L10 yeast polysaccharide supplementation promotes coordinated adjustments in amino acid and nucleotide metabolism, thereby supporting immune function and enhancing physiological resilience under high‐salinity culture conditions or other environmental challenges.

## 5. Conclusion

In conclusion, dietary supplementation with yeast polysaccharide derived from different *Saccharomyces cerevisiae* strains, especially 1 g/kg L10, significantly promoted growth in red tilapia by reducing oxidative stress, moderating inflammatory responses, and strengthening intestinal structure and microbial homeostasis in red tilapia. These coordinated improvements in metabolism, immunity, and gut integrity collectively created an internal environment conducive to efficient growth and overall health. These results highlight the potential of L10 yeast polysaccharide as a promising functional additive for marine aquaculture and offer new insights into its long‐term biological effects under seawater conditions, while the precise molecular mechanisms underlying these effects need further investigation.

## Author Contributions

A special thanks goes to the trial participants who volunteered their time. Zhiyu Zhou and Juyun He designed the experiments, reviewed and edited the manuscript. Ruimin Yuan carried out the rearing work, collected experimental samples, measured experimental parameters, and analyzed the results. Xiaoyi Wu contributed to the writing of the article as part of the study.

## Funding

This work was supported by the Guangzhou Education Bureau University Scientific Research Project (Grant 2024312160).

## Disclosure

Instrumental analyses were performed at the Analytical and Testing Center of Hainan University.

## Ethics Statement

All experiments adhered to the standards outlined in the National Institutes of Health’s (NIH) Guidelines for the Humane Care and Utilization of Laboratory Animals. The study protocol and all associated experimental methodologies received approval from the Experimental Institution Animal Care and Use Committee at Hainan University (Approval Code: HNUAUCC‐2023‐00048).

## Conflicts of Interest

The authors declare no conflicts of interest.

## Data Availability

The data are available upon request from the authors.
